# ^18^F-Fluorocholine PET and Multiphase CT Integrated in Dual Modality PET/4D-CT for Preoperative Evaluation of Primary Hyperparathyroidism

**DOI:** 10.3390/jcm9062005

**Published:** 2020-06-26

**Authors:** Valentin Pretet, Marianela Rotania, Mehdi Helali, Mihaela Ignat, Michel Vix, Alessio Imperiale

**Affiliations:** 1 Nuclear Medicine and Molecular Imaging, ICANS-University Hospitals of Strasbourg, 67033 Strasbourg, France; v.pretet@icans.eu (V.P.); m.rotania@icans.eu (M.R.); m.helali@icans.eu (M.H.); 2 Molecular and Nuclear Medicine, Instituto Oulton, X5000 JJS Cordoba, Argentina; 3 General, Digestive, and Endocrine Surgery, IRCAD-IHU, University of Strasbourg, 67033 Strasbourg, France; danamihaela.ignat@chru-strasbourg.fr (M.I.); michel.vix@chru-strasbourg.fr (M.V.); 4 Faculty of Medicine, University of Strasbourg, 67033 Strasbourg, France; 5 Molecular Imaging-DRHIM, IPHC, UMR 7178, CNRS/Unistra, 67033 Strasbourg, France

**Keywords:** ^18^F-Fluorocholine PET, 4D contrast-enhanced CT, primary hyperparathyroidism, parathyroid, adenoma

## Abstract

The present retrospective study evaluates the diagnostic value of integrated ^18^F-Fluorocholine positron emission tomography/four-dimensional contrast-enhanced computed tomography (^18^F-FCH PET/4D-CT) as second-line imaging in preoperative work-up of primary hyperparathyroidism (pHPT), and compares ^18^F-FCH PET with 4D-CT. Patients with pHPT and negative/discordant first-line imaging addressed for integrated ^18^F-FCH PET/4D-CT were retrospectively selected. Sensitivity and detection rate (DR%) of ^18^F-FCH PET/CT, 4D-CT, and PET/4D-CT were calculated according to the per patient and per lesion analyses, and afterwards compared. Histology associated with a decrease more than 50% of perioperative parathyroid hormone (PTH) blood level was used as a gold standard. Persistent high serum PTH and calcium levels during a 6-month follow-up was considered as presence of pHPT in both operated and non-operated patients. 50 patients (55 glands) were included. 44/50 patients (88%) were surgically treated. On a per patient analysis, sensitivity was 93%, 80%, and 95%, and DR% was 82%, 68%, and 84%, respectively for PET/CT, 4D-CT, and PET/4D-CT. PET/CT was more sensitive than 4D-CT (*p* = 0.046). PET/4D-CT performed better than 4D-CT (*p* = 0.013) but was equivalent to PET/CT alone. On a per gland analysis, sensitivity PET/CT, 4D-CT, and PET/4D-CT was 88%, 66%, and 92%, and DR% was 79%, 57%, and 83%, respectively. PET/CT and PET/4D-CT were more sensitive than 4D-CT alone (*p* = 0.01, *p* < 0.001, respectively). However, PET/CT and PET/4D-CT performed similarly. In conclusion, ^18^F-FCH PET provides better identification of hyperfunctioning parathyroids than 4D-CT and the combination of both did not significantly improve diagnostic sensitivity. Further investigations involving larger populations are necessary to define the role of ^18^F-FCH PET/4D-CT as a “one-stop shop” second-line imaging in preoperative work-up of pHPT, especially considering the additional patient radiation exposure due to multi-phase CT.

## 1. Introduction

Primary hyperparathyroidism (pHPT) is a common endocrine disorder due to excessive secretion of parathyroid hormone (PTH) from one or more hyperfunctioning parathyroid glands. Solitary parathyroid adenoma is the main cause of pHPT. Patients may be asymptomatic or presenting with hypercalcemia-related clinical symptomatology [1,2,3,4]. Surgery is the first-choice treatment with curative intent, particularly in symptomatic patients [3]. Recently, the surgical strategy has evolved from standard bilateral cervical exploration to minimally invasive parathyroidectomy, resulting in a reduction of postoperative complications, operation times, and costs, while maintaining comparable cure rates [5,6,7,8]. This modern approach had increased the demand of preoperative imaging, which is crucial for accurate identification of pathological parathyroids, for embryologic origin characterization (superior vs. inferior parathyroid), and for multiglandular disease assessment [9], three key parameters that inform surgical strategy.

Nowadays, both ^99m^Tc-sestamibi scintigraphy and neck ultrasonography (US) are the first-line preoperative imaging modalities in patients with pHPT [10], although they have suboptimal sensitivity for small and/or ectopic adenomas, particularly in patients with thyroid multinodular goiters [11,12]. Four-dimensional contrast-enhanced computed tomography (4D-CT) and ^18^F-Fluorocholine positron emission tomography/computed tomography (^18^F-FCH PET/CT) appear as two valuable second-line imaging options, superior to ^99m^Tc-sestamibi scintigraphy and neck US [13,14,15,16]. Regarding their relative diagnostic performances, comparisons between ^18^F-FCH PET/CT and 4D-CT have been performed rarely and in a very small number of patients [17,18]. Therefore, no strong evidence exists to give preference to ^18^F-FCH PET/CT over 4D-CT in clinical settings, and the choice of second-line pre-operative investigation is often based on both technical availability and local expertise. In the era of multimodal imaging, the trend is to combine functional and anatomical techniques for disease characterization. In this regard, only one recent study investigated the use of 4D-CT and ^18^F-FCH PET integrated into a single PET/4D-CT examination in patients with pHPT, which demonstrated better diagnostic performances and greater benefits than the use of either positron emission tomography (PET) or 4D-CT alone [19]. However, sensitivity and detection rate of ^18^F-FCH PET were lower than that found in a recent meta-analysis on the subject [14]. It is therefore important to renew the question on the effective clinical value of integrated ^18^F-FCH PET/4D-CT in pHPT, while also considering significant patient radiation exposure related to the multiple phases of 4D-CT scans.

The present study compares ^18^F-FCH PET versus 4D-CT as second-line imaging in pHPT patients and evaluates the potential benefit of integrated PET/4D-CT investigation as a “one-stop shop” diagnostic procedure in preoperative work-up.

## 2. Materials and Methods

### 2.1. Patient Population

This is a non-interventional, monocentric, retrospective study involving patients with pHPT and negative/discordant parathyroid scintigraphy and neck US, who were assessed at the University of Strasbourg Hospital between May 2018 and October 2019 using integrated ^18^F-FCH PET/4D-CT for identification of hyperfunctioning parathyroid glands and preoperative three-dimensional (3D) virtual neck explorations [20,21]. Patients with clinical and biological data missing at the time of PET/4D-CT and with a clinical and biological follow-up less than 6 months after surgery or PET/4D-CT investigation were excluded. A cross-disciplinary team stated about ^18^F-FCH PET/4D-CT indications. In accordance with local institutional guidelines, all patients included gave free and written informed consent for the use of anonymous personal medical data extracted from their file for scientific or epidemiological purposes. 

### 2.2. Imaging Procedures

Neck US and parathyroid scintigraphy were performed according to standard procedures. Several morphological parameters such as lesion diameter, structure, echogenicity, presence of microcalcifications, and intralesional blood flow were evaluated by US. Parathyroid scintigraphy was performed according to standard double isotope ^123^I/^99m^Tc-sestamibi protocol with subtraction technique including neck and mediastinum anterior planar scan (Symbia T2/T6, Siemens Healthcare, Erlangen, Germany, low energy high resolution (LEHR) collimator, 256 × 256 matrix, 300 s per frame) and single photon emission tomography/computed tomography (SPECT/CT) acquisition (LEHR collimator, 128 × 128 matrix, 36 projections of 50 s; helical CT, 140 kV tension, 2.5 mA intensity, without injection of contrast agent). Scintigraphic images were obtained with a simultaneous double window recording, around 2 photopeaks of 5% centered over the 140 keV of ^99^mTc and 159 keV of ^123^I [22].

Integrated ^18^F-FCH PET/4D-CT were performed by a combined PET/CT device equipped with time of flight measurement capacity and a 128 detector-row CT scanner (Biograph128 mCT, Siemens Healthcare, Erlangen, Germany). Imaging protocol integrated:

Dynamic four-phase CT scan, including non-enhanced CT (140 kV, 115m A, 1s per rotation and pitch 0.8, slice thickness of 1 mm) followed by arterial phase (10–15 s after injection, aortic arch threshold >80 HU), venous phase (45 s after injection), and late-venous phase (70 s after injection). 75 mL of iodine contrast agent (Iomeron 400 mg/mL) was intravenously injected with a 2.5–3 ml/s flow rate followed by a saline chaser. CT parameters of arterial and venous phases were: 120 kV, 1 and 15 mAs, 1 s rotation time, pitch 0.8, slice thickness of 1 mm. CT CARE Dose 4D combined with sinogram-affirmed iterative reconstruction (SAFIRE) was used. Diabetic patients withdrew metformin treatment for 2 days after the PET/4D-CT, and abundant hydration was recommended.

^18^F-FCH PET scan. Patients fasted for at least 6 h before 2 MBq/kg of ^18^F-FCH intravenous injection. 10-min step PET acquisition from the mandible to the carina was acquired 60 min after ^18^F-FCH administration in the supine position with arms along the body and headrest. PET datasets were reconstructed iteratively ordered subset expectation maximization (OSEM) algorithm, 2-iterations, 21-subsets) using no contrast-enhanced CT for attenuation correction.

### 2.3. Imaging Analyses

^18^F-FCH PET/CT and 4D-CT from integrated ^18^F-FCH PET/4D-CT were independently interpreted on a dedicated workstation (Syngo.via VB30; Siemens Healthcare, Erlangen, Germany) by a single endocrine radiologist and two nuclear medicine physicians. A third nuclear medicine reviewer was required to reach a consensus when necessary. Referring physicians were aware of patients’ clinical data, biological results, and first-line parathyroid imaging, but were blinded to the results of either PET or 4D-CT scan. Finally, ^18^F-FCH PET/4D-CT studies were interpreted jointly by one nuclear medicine physician and one radiologist.

^18^F-FCH PET and 4D-CT were qualitatively interpreted as positive or negative. Focal non-physiological uptake or tissular nodule (with contrast media enhancement and wash-out of >20 HU at late-venous phase) corresponding to any cervical or thoracic abnormalities discriminable from thyroid tissue and positioned in typical parathyroid sites or in ectopic areas was considered positive. For each positive gland, maximum standardized uptake value (SUVmax), metabolic tumoral volume (MTV), and Hounsfield (HU) value were assessed. The number of pathological findings on ^18^F-FCH PET/CT, 4D-CT, and ^18^F-FCH PET/4D-CT, their topography in reference to the midline and the thyroid gland, ectopic location, and embryologic origin of the parathyroid (superior vs. inferior gland) were recorded.

### 2.4. Gold Standard

Histological proof [23], associated with a decrease of more than 50% of perioperative PTH blood level, as well as PTH and serum calcium values obtained on 6-months follow-up were used as gold standard. In non-operated patients, imaging results were compared to 6-month clinical and biological follow-up after PET/4D-CT, including PTH and calcemia measurements. Persistent high serum PTH and calcium levels were considered to indicate a presence of pHPT.

### 2.5. Statistical Analyses

Results for continuous data were expressed as mean ± standard deviation or median and range as appropriate. Categorical variables were presented as numbers and percentages. Differences between groups were assessed by the Student *t*-test for continuous variables. Sensitivity and detection rate (DR%) of ^18^F-FCH PET/CT, 4D-CT, and PET/4D-CT were calculated according to per-patient and per-lesion analyses. Sensitivity was evaluated on surgically treated patients, while DR% was assessed on the overall population. Moreover, diagnostic performances were determined separately for both identification on the correct side (left/right) and the gland embryological origin using surgical findings as the standard of reference. Patient-to-patient and lesion-to-lesion analyses were performed using the McNemar or Fisher’s exact test as appropriate. The receiver operating characteristic (ROC) curves were used to define the SUVmax threshold for diagnostic purpose. Correlations between variables were assessed using the Pearson correlation coefficient. Two-sided *p* values <0.05 were considered significant. Statistical analyses were performed using open access statistical software (biostatgv.sentiweb.fr, Institut Pierre Louis d’Epidémiologie et de Santé Publique, UMR S 1136, INSERM - Sorbonne Université, Paris).

## 3. Results

### 3.1. Patient Population

In the study period, 90 patients with pHPT and negative/inconclusive first-line imaging were addressed for preoperative evaluation. Contrast media agent injection was contraindicated in 11 patients. An additional 29 cases were excluded because of postponed/refused surgery and missing follow-up data. Thus, 50 patients were finally enrolled (Table 1).

Population median age was 64 years (range 28–79 years), with a female to male ratio of 3:1. 21 (42%) patients were symptomatic. The preoperative mean serum calcium and PTH levels were 2.69 mmol/L and 136 ng/L, respectively. 10 (18%) patients presented with recurrent pHPT. All patients had at least one preoperative imaging modality. 37 (74%), 45 (90%), and 33 (66%) patients had US, parathyroid scintigraphy, or both. 44/50 patients (88%) were surgically treated (*n* = 28, minimally invasive parathyroidectomy; *n* = 15, exploratory cervicotomy including sternotomy in one case; *n* = 1, thoracoscopy) for a total of 51 excised parathyroid glands. Pathology revealed adenoma and hyperplasia in 37 and 13 cases, respectively. One gland was normal (Table 2).

**Figure 1:**
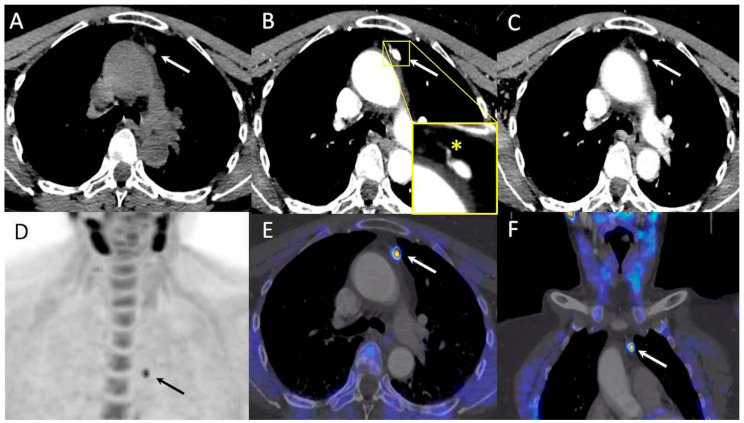
71-year-old woman with primary hyperparathyroidism (pHPT) and symptomatic osteoporosis. Preoperative positron emission tomography/four-dimensional computed tomography (PET/4D-CT) performed after inconclusive neck ultrasonography, identified a mediastinal nodule with high ^18^F-FCH uptake in front of ascending aorta (arrow), with typical contrast media enhancement profile. Hyperplasic parathyroid (0.3 g, 9 mm) was confirmed by pathological analysis. (**A**–**C**): four-dimensional computed tomography (4D-CT), axial slice: no-contrast-media computed tomography (CT), 45 HU; 45 sec post injection, 364 HU (yellow box: polar vessel sign); 70 sec post injection, 180 HU. (**D**): ^18^F-Fluorocholine positron emission tomography (^18^F-FCH PET), anterior maximum intensity projection (MIP); (**E**,**F**): PET/4D-CT, axial and coronal slice.

Surgery located pathological parathyroids as follows: by side, 17 left and 25 right (8 glands were ectopic (Figure 1)); by quadrant, 10 left lower, 7 left upper, 19 right lower, and 6 right upper; by embryological origin: 13 upper and 29 lower glands.

Thirty-nine and five operated patients showed single (SGD) and multiple glandular disease (MGD), respectively. In the remaining six patients (12%) parathyroidectomy was not carried out because of patient refusal, comorbidities, or surgical contraindications. Among them, two revealed biological normalization during six months of follow-up (true negative patient), and four showed persistent pHPT. Considering the prevalence of solitary adenoma in patients with pHPT, we have considered these cases as SGD. Thus, a total of 55 glands in 50 patients have been studied.

### 3.2. Imaging Results

On a per patient analysis, 41 (82%) cases had a true-positive PET/CT, 34 (68%) a true-positive 4D-CT, and 42 (84%) a true-positive PET/4D-CT. A single patient had a false-negative PET/CT and a true-positive 4D/CT, whereas 8 patients had a false-negative 4D-CT and a true-positive PET/CT result. Sensitivity was 93%, 80%, and 95%, and DR% was 82%, 68%, and 84%, respectively for PET/CT, 4D-CT, and PET/4D-CT. PET/CT was more sensitive than 4D-CT (*p* = 0.046), and PET/4D-CT performed better than 4D-CT (*p* = 0.013) but was equivalent to PET/CT alone (Table 3).

**Figure 2:**
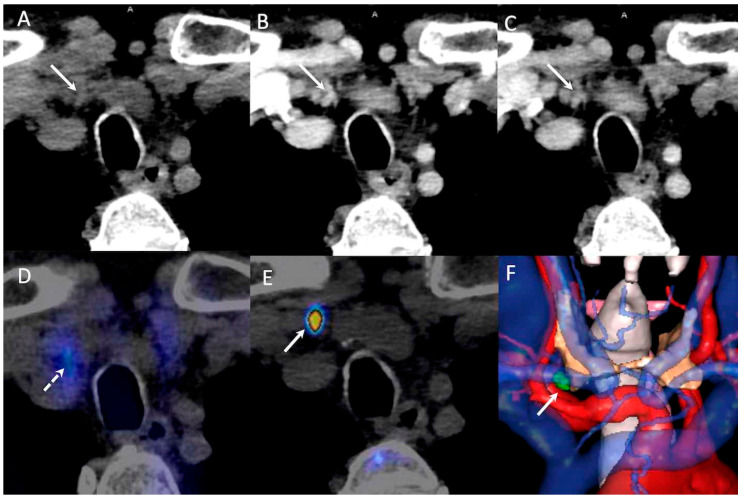
79-year-old man with primary hyperparathyroidism (pHPT) and inconclusive neck ultrasonography (US) and parathyroid scintigraphy. Positron emission tomography/four-dimensional computed tomography (PET/4D-CT) identified one right retro-clavicular nodule with typical contrast-media enhancement profile and high ^18^F-FCH uptake (arrow). Pathology confirmed a lower parathyroid adenoma (0.2 g, 10 mm) descending into the superior mediastinum in the right thyrothymic ligament. (**A**–**C**): four-dimensional computed tomography (4D-CT), axial slice: no-contrast-media computed tomography (CT), 45 HU; 45 sec post-injection, 90 HU; 70 sec post-injection, 63 HU. (**D**): parathyroid scintigraphy, axial slice. (**E**): ^18^F-Fluorocholine positron emission tomography (^18^F-FCH PET), axial slice. (**F**): 3D-virtual neck exploration, anterior view; green: right lower parathyroid, orange: thyroid.

On a per gland analysis, 46/50 hyperfunctioning parathyroids were correctly detected by PET/CT, 34 by 4D-CT, and 48 by PET/4D-CT (Figure 2).

**Figure 3:**
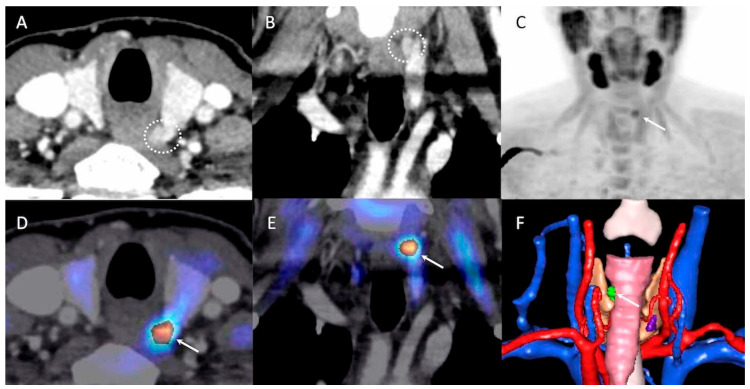
52-year-old woman with recurrent primary hyperparathyroidism (pHPT) and previous history of surgical ablation of left inferior parathyroid adenoma. First-line imaging was negative. Preoperative positron emission tomography/four-dimensional computed tomography (PET/4D-CT) showed increased focal ^18^F-FCH uptake (arrow) corresponding to 1 cm nodule located behind the superior pole of left thyroid lobe (left upper quadrant), with a typical contrast media enhancement (dotted circle). Parathyroid adenoma (0.1 g, 9 mm) was confirmed by pathology. (**A**,**B**): four-dimensional computed tomography 4D-CT, axial and coronal slices; (**C**): ^18^F-Fluorocholine positron emission tomography ^18^F-FCH PET, anterior maximum intensity projection (MIP); (**D**,**E**): PET/4D-CT, axial and coronal slices; (**F**): 3D-virtual neck exploration, posterior view; green: left upper parathyroid, rose: esophagus, orange: thyroid.

PET/CT and 4D-CT were concordant in 39/54 (72%) glands and concordant positive in 32/54 (59%) glands. The two nuclear medicine physicians who independently interpreted the ^18^F-FCH PET imaging were in agreement for 46/50 hyperfunctioning parathyroids (92%), and a third physician was needed to reach a consensus in the remaining 4. There was one false-positive finding common to all three imaging modalities corresponding with a cervical ganglioneuroma, three false-positive results in the same patient common to PET/CT and PET/4D-CT (thyroid remnants after thyroidectomy in close proximity to surgical material, later confirmed by cervicotomy), and five false-positive results of 4D-CT (hyperplasic lymph nodes) exclusively. Hyperfunctioning parathyroids (all glands, adenomas, or hyperplasia) revealed significantly higher SUVmax values compared to non-parathyroid hot-spots (i.e.,: ganglioneuroma and thyroid remnants) (*p* < 0.001, *p* < 0.001, *p* = 0.002, respectively). Considering 2.14 as the SUVmax diagnostic threshold to discriminate hyperfunctioning parathyroids from false positive PET results, sensitivity and specificity were 97.9% and 50% (area under the ROC curve (AUC) was 0.948), respectively (*p* = 0.032). PET-CT missed two pathological glands that were positive on 4D-CT (one adenoma, one hyperplasic gland), whereas 14 hyperfunctioning parathyroids were missed by 4D-CT but correctly identified by PET/CT and PET/4D-CT. Finally, four hyperfunctioning glands were not detected by the three imaging modalities (two adenomas, including one case of oncocytic micro-adenoma, and two hyperplasia). Sensitivity was 88%, 66%, and 92%, and DR% was 79%, 57%, and 83%, respectively for PET/CT, 4D-CT, and PET/4D-CT. PET/CT and PET/4D-CT were more sensitive than 4D-CT (*p* = 0.01, *p* < 0.001, respectively). A statistically significant difference was shown in terms of DR% when PET/4D-CT and 4D-CT (*p* = 0.019) were compared. Both sensitivity and DR% were not significantly different between PET/CT and PET/4D-CT (*p* = 0.48) (Table 3). Considering only the ten included patients with recurrent pHPT (11 pathological glands), sensitivity of PET/CT, 4D-CT, and PET/4D-CT was 100%, 55%, and 100%, and DR% was 85%, 43%, and 85% respectively on a per gland analysis (Figure 3). PET/CT and PET/4D-CT showed equivalent sensitivity and DR%. Moreover, a trend toward significance was observed for sensitivity between PET/CT or PET/4D-CT and 4D-CT (*p* = 0.07).

PET/CT, 4D-CT, and PET/4D-CT correctly lateralized all of the detected pathological parathyroids (left/right side). For embryological origin definition (upper/lower parathyroid), PET/CT and PET/4D-CT had a sensitivity of 97% (one right lower gland wrongly classified as upper gland), and 4D-CT a sensitivity of 81% (one right lower gland misclassified as upper gland, and four right upper glands incorrectly classified as lower glands) (*p* = 0.13).

SUVmax and MTV were significantly higher for adenomas than hyperplasia (*p* = 0.007 and 0.05, respectively), with impact on PET sensitivity (95% vs. 85%). Correlation between SUVmax, MTV, parathyroid UH wash-out, biological patient profile, and glandular pathological features (adenomas vs. hyperplasia) have been investigated. Positive moderate significant correlation was found between MTV and PTH blood level (*r* = 0.51, *p* < 0.001), SUVmax and PTH blood level (*r* = 0.39, *p* = 0.007), MTV and parathyroid gland weight (*r* = 0.36, *p* = 0.038), SUVmax and parathyroid gland weight (*r* = 0.43, *p* = 0.012).

## 4. Discussion

Herein, we have retrospectively evaluated the diagnostic performance of combined stand-alone ^18^F-FCH PET/4D-CT versus both 4D-CT and non-contrast-enhanced PET/CT, as second line imaging for preoperative detection of hyperfunctioning parathyroid glands in 50 patients with pHPT. The main conclusions that can be drawn comprise the following. Firstly, ^18^F-FCH PET/CT was more sensitive than 4D-CT in both per patient (93% vs. 80%, *p* = 0.046) and per gland analysis (88% vs. 66%, *p* = 0.01), confirming the high diagnostic performance of ^18^F-FCH PET/CT as second line imaging in preoperative work-up of pHPT. Secondly, the integration of dynamic 4D-CT and ^18^F-FCH PET in one single PET/4D-CT examination does not seem to offer a significant diagnostic benefit rather than PET alone as shown by a per patient and per gland analysis. Regarding the comparison between ^18^F-FCH PET/CT and 4D-CT, our results are in agreement with the recent study by Piccardo et al. [19], which shows the superiority of PET/CT compared to 4D-CT. However, on a gland-based analysis, PET/CT sensitivity was higher than that reported in Piccardo’s study (80%) and closer to the sensitivity value stated in a recent meta-analysis on the subject (92%) [14]. The design of our study probably contributes to the explanation of these findings. Indeed, 74% of our patients underwent US, 90% had parathyroid scintigraphy, and only 66% had both. This approach could have significantly increased the number of false-negative or inconclusive first-line imaging results, potentially overrating PET/CT sensitivity. Moreover, the potentially different PET/CT devices and related acquisition protocols between our study and those of Piccardo should be considered. In the same study, 4D-CT was more sensitive than was found in our population (74% vs. 66%). The presence of MGD (compared to exclusive SGD) might justify such results [24]. More importantly, and in contrast to Piccardo’s study [19], sensitivities of PET/CT and PET/4D-CT were not statistically different, and the integrated 4D-CT corrected the diagnosis for only two PET-negative hyperfunctioning parathyroids (left lower adenoma of 10 × 5 × 3 mm and 0.1 g, and right superior hyperplasia of 10 × 7 × 1 mm and <0.1 g) in two patients with pHPT and serum PTH values of 94 et 74 pg/mL, respectively. In these two cases, no pathological features contributed to explain the negative PET results, which are probably related to both the size and weight of the gland.

Our investigation does not allow for a “head-to-head” comparison between ^18^F-FCH PET/CT and ^123^I/^99m^Tc-sestamibi SPECT/CT. At this proposal, a recent study underlines the superiority of radiolabeled-choline PET/CT over conventional first-line imaging methods in patients with pHPT [25]. Although some reports suggest superiority of 4D-CT compared to scintigraphy for initial hyperfunctioning gland identification in pHPT [26], 4D-CT is mainly used as a second-line imaging modality in patients with mild hypercalcemia or recurrent hyperparathyroidism (HPT). Comparison between 4D-CT and ^18^F-FCH PET/CT for recurrent pHPT management is available in only one study investigating a population of 20 patients with prior neck surgery for pHPT or unrelated reasons [18]. In this study, a 4D-CT sensitivity was lower than that of ^18^F-FCH PET/CT (63% vs. 96% on a gland-based analysis), suggesting only a confirmatory role for 4D-CT in this challenging population. Our results seem to confirm this observation showing a per gland analysis sensitivity of 55% and 100% for 4D-CT and PET/CT, though the results are not statistically significant (*p* = 0.07), likely because of the small sample size (10 patients, 11 glands). Seven of 50 examined patients had a previously history of thyroidectomy. In these cases, ^18^F-FCH PET showed a sensitivity of 100% for hyperfunctioning parathyroid detection suggesting that previous thyroid surgery has limited consequences on gland detectability. Conversely, a thyroid remnant closer to the surgical clips could have a moderate absorption of ^18^F-FCH resulting in a false positive PET study. In this situation, SUVmax seems useful to discriminate hyperfunctioning parathyroids from other causes of ^18^F-FCH uptake. More studies on larger population are still necessary to confirm the value of (semi)quantitative diagnostic approach.

Four pathologic parathyroids (two patients) were not detected by the three imaging modalities probably due to their small size and weight (less than 10 mm and 0.1 g), an uncommon histological characteristic (one case of oncocytic micro-adenoma), and patient biological profiles (slight PTH increase (83 pg/mL) and subnormal calcemia (2.60 mmol/L) in one case, and moderate PTH increase (102 pg/mL) and vitamin D insufficiency (21 ng/mL) in the other one). Interestingly, some recent evidence points out the potential of a PET dual-time-point acquisition protocol to reduce false negative results in cases of negative/inconclusive standard PET study [27]. As previously reported [28,29], we found a positive and significant correlation between blood PTH level, parathyroid gland weight, and both SUVmax and MTV. Accordingly, PTH blood level could be imagined as a predictor of PET/CT results in preoperative pHPT work-up, but more studies are needed for defining a reliable PTH threshold for clinical use. On the contrary, PET parameters were not related to the kinetic of iodinated contrast-media, as recently reported [19].

We found both SUVmax and MTV significantly higher for adenomas than hyperplasia (*p* = 0.007 and 0.05, respectively) in influencing PET sensitivity. These findings could be explained by previous metabolomic investigations of hyperfunctioning parathyroids [30]. Indeed, adenomas in patients with pHPT and SGD exhibit a higher concentration of choline, phosphorylcholine, and glycerophosphocholine than hyperplastic glands. These metabolites belong to the structural components of cell membranes and their relative abundance probably represents the biologic substrate, justifying the use of ^18^F-FCH PET imaging in pHPT.

4D-CT raises justified concerns for radiation exposure of the patient, which is estimated to be double that of ^99m^Tc-sestamibi scintigraphy [31]. Because 4D-CT scanners use a multi-phase CT protocol, it delivers a high dose to the thyroid, which is problematic in younger patients. Moreover, it needs the injection of contrast medium in a population with potential impaired renal function. Therefore, the benefit of the use of 4D-CT for identifying a symptomatic parathyroid adenoma, particularly if it is possibly resectable, with minimally invasive parathyroidectomy, should be weighed against the risk associated with increased patient radiation exposure. 2D-CT (arterial and venous only without non-contrast phase) could be a good compromise if combined with high resolution US, and it is recommended by some experts to reduce the patient′s radiation exposure while preserving diagnostic accuracy [32].

Recently, PET/MRI hybrid systems have become available for clinical use and may open new research avenues, especially considering the emerging role of magnetic resonance imaging (MRI) in the preoperative identification of hyperfunctioning parathyroid in patients with pHPT [33,34]. However, further studies will be needed to validate and justify the use of this complex imaging approach, including long-term economic considerations. The real challenge to maximize the therapeutic benefit will be to redefine imaging diagnostic protocols according to specific clinical contexts, and to articulate both the strengths and limitations of each diagnostic modality. The optimal combination of imaging technique, imaging sequencing, and the choice of patient needs to be determined to improve the diagnostic accuracy and reduce non-essential patient radiation exposure.

The retrospective character and the lack of pathological gold standard in 6/50 patients represent the main limitations of our study. Second, the relatively small sample size limits a meaningful analysis with potential underestimation of the added value of integrated PET/4D-CT compared to PET alone. Moreover, as previously discussed, the obtained diagnostic performances of ^18^F-FCH PET parathyroid imaging could be potentially influenced by a patient selection bias overestimating PET/CT sensitivity. However, despite these limitations, this study is the largest available that compares ^18^F-FCH PET and 4D-CT, consolidating the potential role of ^18^F-FCH PET as second-line imaging in patients with pHPT. However, the lower availability and higher costs of ^18^F-FCH PET compared to usual first line imaging (US, scintigraphy) may negatively impact routinely clinical utilization.

## 5. Conclusions

^18^F-FCH PET/CT provides better preoperative identification than 4D-CT, and the combination of the two imaging modalities did not significantly improve diagnostic sensitivity in patients with pHPT. However, contrast-enhanced-CT remains useful for operative planning and enables surgical guiding by 3D-virtual neck exploration. Further investigations involving large patient populations are necessary to define the relevance of integrated ^18^F-FCH PET/4D-CT as “one-stop shop” second-line imaging in preoperative work-up of pHPT and to consider the additional patient radiation exposure due to multi-phase CT.

## Figures and Tables

**Table 1:** Patient population characteristics.

	Numbers and Values	Normal Reference
Age (Years), Mean (min–max)	62 (28–79)	
Sex, *n* (%)		
Male	11 (22)	
Female	39 (78)	
PTH (pg/mL), Mean (Range)	136 (44–575)	18.5–88 pg/mL
Calcemia (mmol/L), Mean (Range)	2.69 (2.59–3.26)	2.20–2.60 mmol/L
Phosphoremia (mmol/L), Mean (Range)	0.92 (0.49–1.23)	0.87–1.50 mmol/L
D-Vitamin (ng/mL)	34 (18–69)	>30 ng/mL
Recurrent pHPT, *n* (%)	10 (20)	
Symptoms, *n* (%)		
Asymptomatic	32 (64)	
Osteo-Articular Symptoms	13 (26)	
Nephritic Colic	7 (14)	
Acute Pancreatitis	1 (2)	
History of Thyroidectomy, *n* (%)	7 (14)	
Full	2 (4)	
Partial	5 (10)	
Thyroid Disease		
Multinodular Goiter	5 (10)	
Active Graves’ Disease	1 (2)	
Calcimimetics, *n* (%)	5 (10)	
MEN1, *n* (%)	1 (2)	
First-Line Imaging, *n* (%)		
Negative	21 (42)	
Discordant	29 (58)	

PTH: parathyroid hormone; pPTH: primary hyperparathyroidism; MEN: multiple endocrine neoplasia.

**Table 2:** Characteristics of hyperfunctioning parathyroids from 44 operated patients.

	Numbers and Values
Location, *n* (%) Right Upper Right Lower Left Upper Left Lower Ectopic *	50 6 (12) 19 (38) 7 (14) 10 (20) 8 (16)
Histology, *n* (%) Adenoma Hyperplasia	50 37 (74) 13 (26)
Weight (g), Mean (min–max)	0.41 (0.10–1.70)
Size (mm), Mean (min–max)	14.6 (5–55)

* Esophageal attachment (*n* = 4), upper and anterior mediastinum (*n* = 2), ahead of thyroid gland (*n* = 1), pharyngeal attachment (*n* = 1).

**Table 3:** Head-to-head comparison between PET/CT, 4D-CT, and PET/4D-CT in the whole population including 50 patients and 54 glands.

	PET/CT	4D-CT	PET/4D-CT	PET/CT vs. 4D-CT	PET/CT vs. PET/4D-CT	4D-CT vs. PET/4D-CT
**Sensitivity**						
**Per Patient**	39/42 (93%)	33/40 (80%)	40/42 (95%)	*p* = 0.046	*p* = 1	*p* = 0.013
**Per Gland**	44/50 (88%)	33/50 (66%)	46/50 (92%)	*p* = 0.01	*p* = 0.48	*p* < 0.001
**Detection Rate**						
**Per Patient**	34/50 (68%)	34/50 (68%)	42/50 (84%)	*p* = 0.23	*p* = 1	*p* = 0.11
**Per Gland**	46/58 (79%)	34/60 (57%)	48/58 (83%)	*p* = 0.066	*p* = 0.48	*p* = 0.019

PET/CT: positron emission tomography/computed tomography; 4D-CT: four-dimensional computed tomography; PET/4D-CT: positron emission tomography/four-dimensional computed tomography.
